# Are Patient Self-reported Healthcare Utilization Data Reliable in Persons With IBD?

**DOI:** 10.1093/crocol/otab033

**Published:** 2021-06-12

**Authors:** Wael El-Matary

**Affiliations:** Section of Pediatric Gastroenterology, Department of Pediatrics, Max Rady College of Medicine, Rady Faculty of Health Sciences and Children’s Hospital Research Institute of Manitoba, Winnipeg, Manitoba, Canada

In its 2018 impact report, Crohn’s and Colitis Canada (CCC), the significant health and economic burden of inflammatory bowel disease (IBD) was highlighted.^1^ Over the last 2 decades, all stakeholders have been working together to improve quality of care provided to persons with IBD and reduce the negative impacts of this serious disease. Several quality improvement programs have been successfully created with very promising results.^2,3^ One great example of quality improvement initiative is the ImproveCareNow (ICN) pediatric network that includes over 100 pediatric IBD centers mainly in the United States but also internationally.^4^ Another collaborative work to improve learning health systems (LHS) is the Crohn’s and Colitis Foundations’ IBD Qorus LHS.^5^ This is a network of over 50 academic and private IBD practices that work together toward improving healthcare outcomes in adults with IBD and have already lead to reductions in emergency room visits, hospitalizations, and opioid use in adults with IBD.^6^

In any of these networks, and to allow clinicians and administrators to improve the healthcare system, collecting accurate data on the provided services, quality measures, and healthcare outcomes is prudent for the success of LHS. Several data sources exist and include administrative databases, electronic health records (EHRs), and patient self-reported data with pros and cons in each option.

In this issue of Crohn’s & Colitis 360, Van Deen et al^7^ compared patient self-reported data on medication utilization, vaccinations, and some other aspects of healthcare use such as emergency department visits and hospitalization, to EHR within 4 sites of the IBD Qorus LHS. They analyzed data from 328 IBD patients who were surveyed about utilization of some healthcare services, mainly IBD-related emergency department visits, hospitalizations, and computerized tomography scans that they received in 6-month period before the survey. The agreement between patient-reported and EHR-obtained data was reassuring and ranged from 89% to 96%. At the time of patients’ visit, the agreement between self-reported medication use and the EHR was 92% for both corticosteroid and opioid use. On the other hand, the agreement between self-reported and EHR-reported vaccination status was 61% agreement for influenza vaccinations in the previous year and 68% agreement for pneumococcal vaccinations that were ever received. The study results are important and concordant with other studies that showed that, despite a relatively long recall period, patient self-reported data on hospital utilization and medication are usually accurate and reliable.^8,9^ However, and as mentioned by the authors, the study conclusions are limited by the lack of the exact survey completion rate and hence the probability of selection bias.

Overall, this work is a confirming step on the reliability of patient-reported data and clearly signifies the importance of partnership between patients and healthcare personnel toward improving healthcare systems.

## Data Availability

NA as this is an editorial article.
